# Book Reviews

**Published:** 1885-05

**Authors:** 


### Book Reviews.

Transactions of the Thirty-Fourth Annual Meeting of the Illinois State Medical Society, held at Chicago, May 20, 21 and 22, 1884.

The Transactions of the Thirty-fourth Annual Meeting of the Illinois State Medical Society is an interesting and valuable volume. The papers are numerous, important, and exhibit more than the usual amount of original investigation. Professor H. A. Johnson's Case of "Bilateral Paralysis of the Abductor of the Vocal Chords" will attract general attention, as a clear, distinct exposition of a relatively rare pathological condition. The case was seen in London by Morrell Mackenzie and Lennox Brown, who concurred in Professor Johnson's diagnosis and treatment.

Professor Christian Fenger contributes two important papers, "Excision of Hip and Knee Joints, with Exhibition of Patients," and "On Surgical Treatment of Gangrene of the Lung."

Professor Fenger's views, on these subjects, are so well known to the profession that it is not necessary to allude to them in detail. Unfortunately, there was no discussion of the subject matter of Professor Fenger's paper,—a fact to which Dr. E. P. Cook, of Mendota, alluded in well chosen terms at the time. A paper on "Vaccination," presenting facts known for an indefinite period of time, on the other hand, was sufficient to provoke a very lively debate.

Professor D. W. Graham's paper, entitled, "A Case of Goitre with Obstruction," is a valuable contribution to the literature of the subject.

Professor Charles Warrington Earle's paper, entitled "Four Cases of Pancreatic Anæmia," was one of the most interesting contributions to the department of internal medicine.

The members of the Committee on Publication deserve much credit for the selection of papers for publication, and for the many technical excellencies of the volume. W. W. J.

---

THE STORY OF MY LIFE. *By* J. MARION SIMS, M. D., LL.D. *Edited by his Son*, H. MARION-SIMS, M. D. *New York:* D. APPLETON & CO. 1884. *12mo., pp. 471.*

As an autobiography, "The Story of My Life" is a unique book. It reads like a romance. With characteristic simplicity and guilelessness, the great clinical surgeon has given to the profession, and the world at large, a graphic portraiture of the evolution of a South Carolina lad into one of the greatest modern benefactors of his race.

"Mine," says he, "has been a real romance, full of incident, anxiety, hope and care; some disappointments, and many successes, with much sickness and sorrow; but it has also been full of joy, contentment, and real happiness."

A priceless heritage, "The Story of My Life" deserves a place on the same shelf with "Plutarch's Lives." As a vivid picture of contemporaneous medical history, American and European, the value of the book is great. With a keen analytical eye, Sims has drawn pen-sketches, with critical accuracy, of the modern heroes of medicine. Simpson, Nélaton, Trousseau, Civiale, Ricord, Chassaignac, Larrey, Huguier, Velpeau, Baker Brown, Malgaigne, Thompson, Erichsen and others have assumed living forms under the influence of his magic pen.

Finally, he has magnanimously shielded from public scrutiny the anomalous conduct of his New York *confrères*, in connection with the establishment and perpetuation of the Woman's Hospital.

The reviewer's task is light. Every member of the medical profession will appreciate the book, and many will possess it.

Already it has been translated into foreign languages, and the first German edition is nearly exhausted.

The book is marred by an injudicious introduction of twenty-eight pages, from the pen of an intimate friend. The statement is made in this introduction that Sims, in 1863, was authoritatively recognized as "the foremost clinical surgeon of the age." As a contemporary of v. Langenbeck, Billroth, Péan, Lister, Volkmann, König, Esmarch, Wells, Keith, Paget, Erichsen, Thompson, Gross, Bigelow, the elder Pancoast, Nélaton, the statement appears remarkable.

The harsh sectional allusion to General W. Tecumseh Sherman,—"That commander bore among his baptismal titles, as if in forecast of his military career, the name of a sanguinary Indian savage,"—is irrelevant, in bad taste, and opposed to Sims' own views upon the subject.

In connection with Sims' speculum, Dr. Emmet's very remarkable and extravagant statement is quoted:

"From the beginning of time to the present, I believe that the human race has not been benefited to the same extent, and within a like period, by the introduction of any other surgical instrument."

W. W. J.

HAND BOOK OF THE DIAGNOSIS AND TREATMENT OF SKIN DISEASES. *By* ARTHUR VAN HARLINGEN, M. D. *Professor of Diseases of the Skin in the Philadelphia Polyclinic and College of Graduates in Medicine, etc., etc. With two colored plates. Philadelphia:* BLAKISTON, SON & CO. 8 vo., *pp. 282.*

The author of this little manual has become well known to the profession by his previous valuable contributions to Dermatological literature. Among the most important of these may be named a full description of the medicinal eruptions, which appeared in the *Archives of Dermatology*, in Oct. 1881; a concise chapter on diseases of the skin, in the American edition of Holmes' System of Surgery; together with papers on scleroderma, the pathology of serborrhœa, etc., which have from time to time appeared over his signature in print. By education and experience, the author is well fitted for his task.

In the limits of a small volume like the present, one can look for but little beside a compendious statement of what is known and accepted on the subjects considered. Indeed, the demand is for scrupulous omission of all points open to serious discussion. As a result, the pages before us are well adapted to the needs of those for whom the work was probably intended, viz: busy practitioners and students who either can not or will not consult the larger treatises. For the sake of convenience, the names of all diseases of the skin are here arranged in alphabetical order, with references by which each can be readily assigned to its place in the nine chief divisions recognized in the Hebra classification and adopted by the American Dermatological Association. It is proper to note in passing, however, that at its late meeting the American Dermatological Association reduced the number of these classes to eight, by the omission of that which originally included

"ulcers." When it is considered that an ulcer always is the result of a precedent pathological process, the reason for such a modification in the interests of a simple and scientific classification becomes apparent.

Dr. Van Harlingen's hand-book is decidedly in advance of all its competitors among the smaller manuals, in point of practical suggestiveness. It is well named a hand-book of "Diagnosis and Treatment." Eczema in its protean characters furnishes always the best test of an author's ability to successfully meet the exigencies of practice; and upon this subject the therapeutical hints given in the book before us, are eminently valuable. About one-seventh of the volume is devoted exclusively to the treatment of the several varieties of eczema, and it would be difficult to find in any work a safer and more trustworthy exposition of the several intricate problems presented in therapeutic management of that disease.

The two colored plates can scarcely be said to add much to the value of the manual. In other respects, the typographical appearance of the pages is all that could be desired.

J. N. H.

---

A Text Book of Hygiene: A Comprehensive Treatise on the Principles and Practice of Preventive Medicine, from an American Standpoint. *By* George H. Rohe, M. D., *Professor of Hygiene, College of Physicians and Surgeons, Baltimore, etc. 8vo., cloth, pp. xv., 324. Baltimore:* Thomas & Evans. 1885.

This little handbook, printed in large type, will probably find its way into the hands of a multitude of readers, among the laity as well as the profession. It treats of the following: Air, Water, Food, Soil, Sewage, Habitations, Hospitals, Schools, Occupation, Military and Marine Hygiene, Prison

Exercise, Bathing, Clothing, Disposal of the Dead, Contagious and Epidemic Diseases, Antiseptics and Disinfectants, Quarantine and Vital Statistics. Although each of these subjects is treated of in a concise manner, the bibliography at the end of each chapter will render more extensive study very easy. The clear, intelligent style of the author will be especially appreciated by popular readers, and it is to be hoped that this will be followed by many and more extensive editions. The weak points and deficiencies of the book partake of the hygienic ignorance of the times, made more manifest by the rapid advance of architecture, commerce, and the requirements of political economy, and will disappear as experimental hygiene is more thoroughly tested. H. D. V.

